# 610. Voriconazole versus isavuconazole in patients with invasive aspergillosis: a retrospective study in a medically insured population across the United States

**DOI:** 10.1093/ofid/ofad500.676

**Published:** 2023-11-27

**Authors:** Sophea Chan, Laura Leigh Stoudenmire, Duna Zhan, Xianyan Chen, Andrés F Henao Martínez, Daniel B Chastain

**Affiliations:** Vanderbilt University Medical Center , Nashville , Tennessee; Phoebe Putney Memorial Hospital, Albany, Georgia; dunaz@uga.edu, Athens, Georgia; UGA Franklin College of Arts and Sciences, Athens, Georgia; University of Colorado Anschutz Medical Campus, Aurora, CO; University of Georgia College of Pharmacy, Albany, Georgia

## Abstract

**Background:**

Isavuconazole demonstrated similar outcomes but improved tolerability versus voriconazole (VCZ) for invasive aspergillosis (IA). Despite this, VCZ remains the treatment of choice for IA. Therefore, we compared outcomes between patients with IA treated with VCZ versus isavuconazole (ISA).

**Methods:**

Patients with IA treated with either VCZ or ISA as monotherapy between January 1, 2017 and August 31, 2020 were identified from the IBM® MarketScan® Research Databases using ICD-10-CM diagnosis codes and national drug codes (NDC), respectively. The index date was the first fill date for either VCZ or ISA during the study period. To be included, patients were required to have continuous medical and pharmacy benefits enrollment before the index fill for either VCZ or ISA, throughout the treatment period, and for ≥ 28 days after treatment ended based on day supply. Patients with a history of liver transplant or hepatic dysfunction were excluded. Treatment completion, defined as fill history for ≥ 42 days, and adverse events, during the treatment and follow-up periods, were compared between those treated with VCZ and ISA.

**Results:**

Of 563 patients with IA, 425 received VCZ and 138 received ISA. The mean age (51.4 ± 16.8 vs 51.9 ± 12.8 years, p=0.77) and proportion of male patients (52% vs 49%, p=0.61) were similar in both groups. The most common comorbidities among those treated with VCZ and those treated with ISA were chronic obstructive pulmonary disease (38% vs 27%, p=0.03), diabetes mellitus (22% vs 22%, p=0.98), and lymphoma (8% vs 14%, p=0.04). Similar proportions of patients in the VCZ group and ISA group had commercial insurance or Medicare (63% and 10% vs 71% and 12%, p=0.94 overall). Diagnosis of IA was evenly distributed between the inpatient and outpatient settings in each group. The rate of treatment completion was comparable between patients prescribed VCZ and those prescribed ISA (88% vs 91%, p=0.50). Adverse events were reported by 53% of patients treated with VCZ and 45% treated with ISA (p=0.11), though few patients switched therapy during the 6-week treatment period (n=4 for VCZ and n=1 for ISA).

**Conclusion:**

VCZ was prescribed 3 times more often than ISA for IA, but rates of treatment completion and adverse events were similar between groups.

**Disclosures:**

**All Authors**: No reported disclosures

